# Limb symmetry during double-leg squats and single-leg squats on land and in water in adults with long-standing unilateral anterior knee pain; a cross sectional study

**DOI:** 10.1186/s13102-017-0085-x

**Published:** 2017-12-11

**Authors:** Anna C. Severin, Brendan J. Burkett, Mark R. McKean, Aaron N. Wiegand, Mark G. L. Sayers

**Affiliations:** 1 School of Health and Sports Sciences, University of the Sunshine Coast, Sippy Downs Drive 90, Sippy Downs, QLD 4556 Australia; 2 School of Science and Engineering, University of the Sunshine Coast, Sippy Downs Drive 90, Sippy Downs, QLD 4556 Australia

**Keywords:** Inertial sensors, Asymmetry, Kinematics, Aquatic exercise

## Abstract

**Background:**

The presence of pain during movement typically results in changes in technique. However, the physical properties of water, such as flotation, means that water-based exercise may not only reduce compensatory movement patterns but also allow pain sufferers to complete exercises that they are unable to perform on land. The purpose of this study was to assess bilateral kinematics during double-leg squats and single-leg squats on land and in water in individuals with unilateral anterior knee pain. A secondary aim was to quantify bilateral asymmetry in both environments in affected and unaffected individuals using a symmetry index.

**Methods:**

Twenty individuals with unilateral knee pain and twenty healthy, matched controls performed body weight double- and single-leg squats in both environments while inertial sensors (100 Hz) recorded trunk and lower body kinematics. Repeated-measures statistics tested for environmental effects on movement depths and peak angles within the anterior knee pain group. Differences in their inter-limb symmetry in each environments was compared to the control group using analysis of variance tests.

**Results:**

Water immersion allowed for greater movement depths during both exercises (double-leg squat: +7 cm, *p* = 0.032, single-leg squat: +9 cm, *p* = 0.002) for the knee pain group. The double-leg squat was symmetrical on land but water immersion revealed asymmetries in the lower body frontal plane movements. The single-leg squat revealed decreased hip flexion and frontal plane shank motions on the affected limb in both environments. Water immersion also affected the degree of lower limb asymmetry in both groups, with differences also showing between groups.

**Conclusions:**

Individuals with anterior knee pain achieved increased squat depth during both exercises whilst in water. Kinematic differences between the affected and unaffected limbs were often increased in water. Individuals with unilateral anterior knee pain appear to utilise different kinematics in the affected and unaffected limb in both environments.

## Background

Anterior knee pain (AKP) is an umbrella term for pain around the anterior aspects of the knee that is aggravated by physical activity [1] and common tasks in daily life such as descending stairs and squatting [2]. It is one of the most common conditions presenting in physiotherapy clinics [1, 3], and may present as a unilateral or bilateral condition [4]. AKP has been linked to lower body malalignments and deficits in strength, flexibility, and neuromuscular function [5]. Prolonged pain has been suggested to change muscular function and disrupt inter-muscular coordination [6], so it is not surprising that previous research has reported compromised muscle functions in individuals with AKP [2, 7]. Similarly, research indicates that these individuals employ compensatory movement strategies during exercises like single-leg squats (SLS) and running [8, 9]. Common strategies include increased pelvic obliquity, lateral trunk lean, and valgus alignment [10], which probably contributes to the continued aggravation of AKP [2, 3, 9].

Rehabilitation programs often target hip and gluteal function and include double-leg squats (DLS) and SLS to improve strength, balance, and coordination [1, 5]. Despite AKP frequently presenting unilaterally [4], most biomechanical studies compared affected individuals with healthy controls and failed to discuss bilateral differences [8, 9]. This is troubling, as research has reported bilaterally different kinematics following unilateral knee injuries [4, 7, 11, 12]. It is likely that long-standing unilateral AKP also result in bilaterally asymmetrical kinematics, and further examinations are needed to map compensatory movements.

Water-based rehabilitation is anecdotally effective for AKP, and although previous research supports its application for rehabilitating degenerative knee conditions [13], research on its efficacy on AKP is limited. The aquatic environment reduces loading [14, 15], improves strength [16, 17], and supports balance [18, 19], thus providing a suitable alternative to land-based rehabilitation for AKP. Aquatic therapy is also known to reduce pain and increase range of motion [16, 20], which are important benefits for rehabilitation [1]. Importantly, previous research has highlighted that water immersion encourages different kinematics compared to land due to buoyancy, viscosity, and density [15, 20, 21]. Particularly, water-based squat tasks portrayed increased movement depths and different trunk and lower body kinematics compared to squats performed on land [20]. Previous research has not quantified kinematic impacts of water immersion on individuals with AKP. Such information would be useful for practitioners when programming for water-based rehabilitation.

Bilateral differences in kinematics are often quantified in injured populations as their kinematics can reflect compensatory movements, and affect the efficacy of rehabilitation programs [11]. Few published reports have assessed asymmetry in water, but a recent analysis highlighted increased asymmetries in water for healthy individuals during gait [22]. Despite only assessing spatiotemporal implications, the authors highlighted that symmetry can provide important insights into movement control. No published research has quantified kinematic asymmetries during DLS and SLS between land and water at the time of submission.

Traditionally, symmetry index (SI) calculations rely upon discrete data and are not applicable to time series data [23, 24], but this issue was addressed by Nigg, et al. [24] who developed an SI calculation for continuous data sets. This method has not been used to quantify bilateral asymmetry in individuals with AKP compared to healthy controls. An increased understanding of the effects of water immersion on symmetry in individuals with AKP would clarify the roles of aquatic therapy for rehabilitation further.

Accordingly, this study aimed to assess kinematic implications of water immersion on individuals with AKP during DLS and SLS by (1) quantifying differences in frontal and sagittal plane peak joint and segment angles and, (2) compare the environmental impacts on bilateral asymmetry with healthy controls. It was hypothesised that individuals with AKP would utilise different kinematics in water than on land, and that water immersion would increase the degree of asymmetry in this population compared to the uninjured control group.

## Methods

### Participants

Twenty young adults with chronic AKP (10 males and 10 females) and 20 healthy age- and gender-matched adults volunteered for participation (AKP group 22.8 ± 4.0 y, 71.2 ± 13.0 kg, 1.72 ± 0.09 m, control group 22.2 ± 2.9 y, 67.6 ± 13.4 kg, and 1.72 ± 0.10 m). The AKP group reported unilateral pain for at least three months (3–48 months) but were otherwise healthy. All participants were physically active and had at least three years’ experience with body weight exercises, and no prior exposure to water-based exercise. Self-reported leg dominance was determined as the participants’ preferred kicking leg (right: 18, left: 2 in each group). In accordance with the Human Research Ethics Committee approval, any participant with knee pain during stair descent was excluded from participation. Informed written consent was obtained before testing.

### Experimental design

**Fig. 1:**
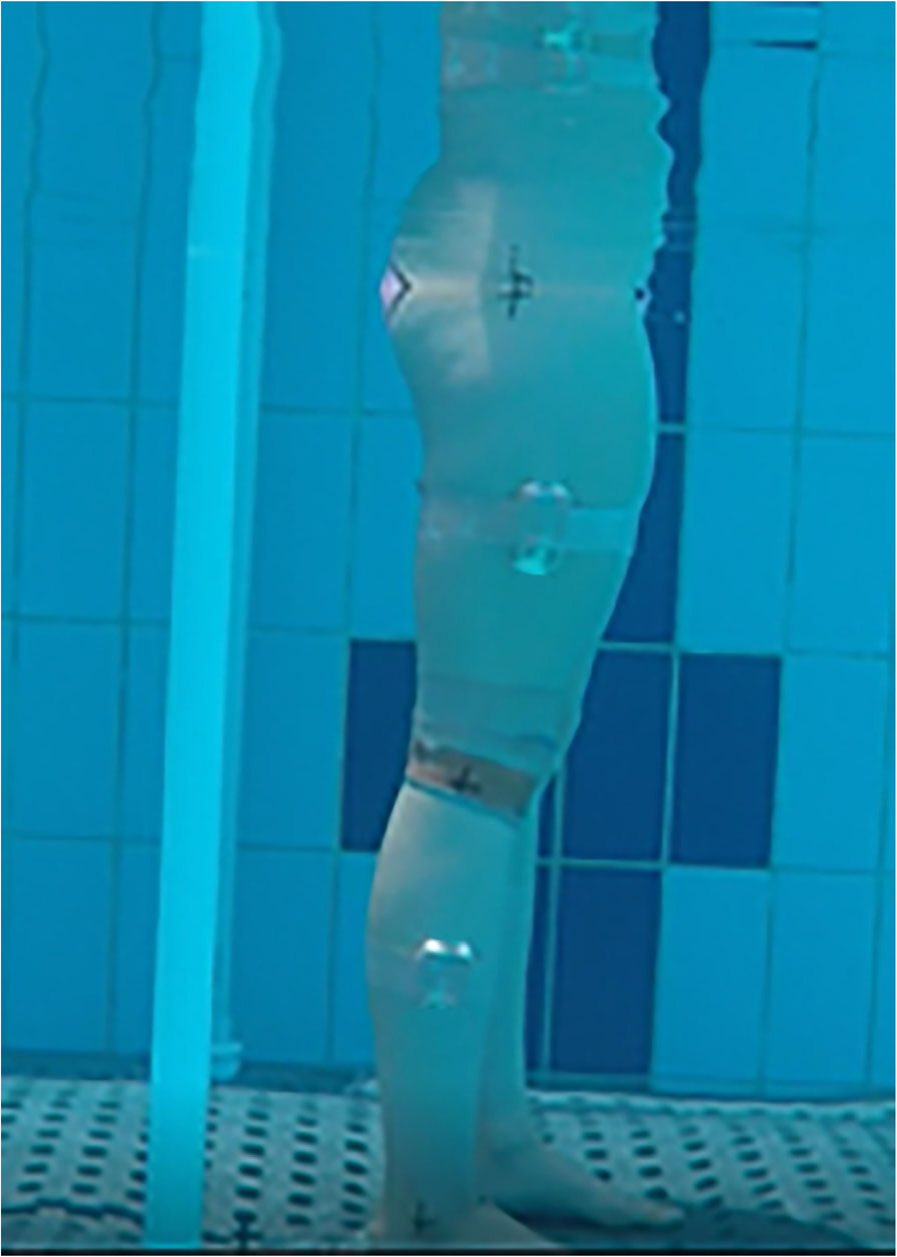
Photograph showing the set-up during the water-based session

This study used inertial sensors, which have successfully been used to record underwater sagittal and frontal plane kinematics [20, 21]. Four sensors (100 Hz) (Nanotrak, Catapult sports, Docklands, VIC) were allocated bilaterally to the lateral thighs and shanks, halfway between the proximal and distal joint centres (Fig. 1). One sensor was positioned over the third thoracic vertebra and another was attached to the sacrum. To ensure consistency in sensor allocation, the same person attached the sensors at each testing occasion. A ten-second static calibration was performed before each exercise in the anatomical position to establish 0^o^ orientations for the sensors [21]. To avoid intra-sensor bias, the sensor allocations were consistent throughout testing.

Each participant attended two testing occasions; the first in a motion laboratory and the second at a pool complex within one week of the first session. A platform of adjustable height ensured a water depth to the greater trochanter on each participant (87 ± 5 cm). The Olympic standard pool had a water temperature of 29.1 °C ± 1.0 during the testing.

Both sessions started with a self-selected warm up of two to three minutes of aerobic activity and five to ten practice repetitions of the exercises for familiarization [18], followed by ten DLS and ten SLS on each leg. During the SLS, the contralateral limb was flexed at the knee to 70–90° and positioned behind the body. The arms were maintained outstretched in front during both exercises. No instructions were provided concerning stance width and squat depth [11], and the tempo was dictated by a metronome (100 bpm). The participants completed one repetition over eight beats, four to descend, and four to ascend. Two minutes’ rest was allowed between the exercises, and no randomization was used to allow the same task familiarization for each participant.

### Data processing

The raw data from the ten repetitions were smoothed with a custom, variable-width, non-weighted box-smoothing algorithm and the slope for any internal drift was quantified using linear regression and subtracted. A more in-depth description of the data processing can be found in Severin, et al. [20]. The smoothed data were integrated to yield segmental displacements and the ten repetitions were identified based on peak sagittal plane angles. The segmental angles from the shank, thigh, sacrum, and trunk were used to calculate the relative angles [21], which was done using the following equations:

1 $$ {\theta}_{knee}={\theta}_{shank sensor}+\left(180-{\theta}_{thigh sensor}\right) $$

2 $$ {\theta}_{hip}={\theta}_{sacral sensor}+\left(180-{\theta}_{thigh sensor}\right) $$

3 $$ {\theta}_{trunk}={\theta}_{thoracic sensor}+\left(180-{\theta}_{sacral sensor}\right) $$

To allow comparisons between the individual sensors, and to calculate joint angles, all data were adjusted to comply with standard Euler conventions, with flexion, adduction, and internal rotation portrayed as positive rotations [25]. The data were time normalized to 1000 data points in order to simplify comparisons. Data from the sacral sensor determined the vertical displacement of the pelvis to indicate squat depth [26]. The variables of interest included bilateral peak sagittal angles and SI-scores of the shank, thigh, and thorax segments as well as knee, hip, and trunk angle.

### Data analysis

This study followed the convention of limiting analyses to the sagittal and frontal planes [20, 21] due to questioned accuracy of internal sensors in the transverse plane [27]. Statistical analyses were performed on all kinematic variables using IBM SPSS version 22 (IBM, New York, NY). Bilateral differences in kinematics in the AKP group were assessed by comparing peak angles for segments and joints between environments. The SI-scores determined bilateral asymmetry between the affected and unaffected limb in the AKP group, and between the dominant and non-dominant limb in the control group. An SI score of zero-score indicated perfect symmetry [24]. The SI score was calculated with the calculation used by Nigg, et al. [24]:

4 $$ SI=\underset{t={t}_1}{\overset{t_2}{\int }}A\mid {x}_r(t)-{x}_1(t)\mid dt $$

5 $$ A=\frac{2}{range\left({x}_r(t)\right)+ range\left({x}_1(t)\right)} $$

Where the value of a specific variable at the time (*t*) for the right limb is represented by *x*_*r*_(*t*), and *x*_*l*_(*t*) represents the same variable for the left limb.

The movement depths between environments were tested for covariance and the kinematic variables were tested for compliance with the assumptions of an analysis of covariance. Wherever the assumptions were met, an analysis of covariance determined significant differences between the environments, and elsewhere, a Wilcoxon Singed-rank test was used. The SI-scores for both groups were tested for normality using a Shapiro-Wilk’s tests, and whenever it was violated, a Mann-Whitney U test was used to determine differences between groups. Where normality was indicated, an analysis of variance was used to test for between-group differences in SI- scores. Effect sizes were calculated and ranked using the method developed by Cohen [28], with scores d > 0.2 considered small, >0.5 moderate and >0.8 considered large effect. The alpha level was set at *p* < 0.05.

## Results

The analysis showed that water immersion affected the maximal depth for the AKP group both during the DLS (land: 33 ± 8 cm, pool: 40 ± 11 cm, *p* = 0.032, d = 0.70) and the SLS (affected limb: land: 20 ± 7 cm, pool: 29 ± 10 cm, *p* = 0.002, d = 1.06, unaffected limb: land: 19 ± 6 cm, pool: 27 ± 9 cm, *p* = 0.003, d = 1.00). Participants in the AKP group verbally reported that water immersion reduced any sensation of pain or discomfort during both exercises.

The analysis revealed that the limbs reached different peak angles in the two environments during the exercises, although it was more evident during the SLS. Water immersion increased the frontal plane peak angles of the affected limb during the DLS, but did not affect it sagittal plane motions (Table 1). The unaffected limb did not show any statistically significant differences between the environments in either plane of motion. For the SLS, water immersion increased the sagittal plane peak angles of both limbs, and decreased those of the thorax segment and trunk angle (Table 2). The changes in the frontal plane were less congruent during the SLS, as some peak angles increased, while others decreased or remained unaffected by immersion. Similarly, the kinematic differences between the limbs generally increased in the sagittal plane, whereas the differences in the frontal plane were less consistent.

**Table 1:** Peak angles (±SD) for double-leg squats between the limbs of the AKP group in both environments

	Land			Pool		
	Unaffected	Affected	*d*	Unaffected	Affected	*d*
Shank angle (°)	21.3 ± 8.0	21.8 ± 8.0	0.05	18.3 ± 8.4	22.0 ± 6.3	0.50
Thigh angle (°)	59.6 ± 27.9	62.6 ± 22.6	0.12	65.4 ± 22.5	68.9 ± 21.9	0.16
Knee flexion (°)	94.4 ± 16.7	90.2 ± 19.5	−0.23	95.2 ± 10.4	91.6 ± 13.4	−0.27
Hip flexion (°)	73.8 ± 17.0	71.7 ± 31.1	−0.08	77.8 ± 19.7	75.5 ± 15.8	−0.10
Shank medial deviation (°)	9.2 ± 5.3	8.2 ± 5.7	−0.20	10.0 ± 5.0	11.9 ± 4.2^b^	0.42
Thigh lateral deviation (°)	10.3 ± 8.5	13.6 ± 9.4	0.37	12.4 ± 10.4	20.6 ± 9.0^b^	0.84*
Hip adduction (°)	6.0 ± 8.5	3.6 ± 5.6	−0.33	4.1 ± 3.8	3.0 ± 3.1	−0.32
Knee adduction (°)	17.2 ± 12.8	20.0 ± 13.7	0.21	19.8 ± 13.0	30.3 ± 11.6^a^	0.85*
Hip abduction (°)	12.0 ± 9.0	12.7 ± 9.9	0.07	10.8 ± 8.0	18.8 ± 10.7^b^	0.85*
Knee abduction (°)	3.8 ± 3.1	2.1 ± 2.5	−0.58	3.4 ± 2.8	3.7 ± 3.7	0.08

*indicates significant difference between limbs at *p* < 0.05

^a^indicates large within-limb effect size between environments at Cohen’s *d* > 0.8

^b^indicates moderate within-limb effect size between environments at Cohen’s *d* > 0.5

**Table 2:** Peak angles (±SD) for single-leg squats between the limbs of the AKP group in both environments

	Land			Pool		
	Unaffected	Affected	Cohen’s *d*	Unaffected	Affected	Cohen’s *d*
Shank angle (°)	26.0 ± 10.0	24.0 ± 5.8	−0.25	25.8 ± 8.4	27.8 ± 7.7^b^	0.25
Thigh angle (°)	36.1 ± 14.7	36.2 ± 11.8	0.00	51.3 ± 9.0^a^	49.1 ± 8.6^a^	−0.25
Thorax angle (°)	23.8 ± 11.5	23.2 ± 9.8	−0.06	17.9 ± 11.2^b^	16.2 ± 8.6^b^	−0.16
Knee flexion (°)	65.0 ± 13.5	61.6 ± 10.4	−0.28	78.1 ± 13.4^a^	76.0 ± 12.1^a^	−0.17
Hip flexion (°)	42.8 ± 12.9	33.7 ± 11.4	−0.75*	59.8 ± 11.3^a^	42.5 ± 9.6^a^	−1.37*
Trunk flexion (°)	20.6 ± 13.4	19.6 ± 11.2	−0.08	21.1 ± 9.4	12.4 ± 7.4^b^	−1.03*
Shank medial deviation (°)	2.4 ± 2.3	7.5 ± 4.8	1.35*	10.4 ± 6.7^a^	11.3 ± 7.4^b^	0.13
Thigh lateral deviation (°)	5.2 ± 4.1	6.3 ± 5.7	0.22	8.7 ± 8.5^c^	9.7 ± 7.3^b^	0.13
Thorax lateral deviation (°)	5.0 ± 6.0	3.5 ± 2.9	−0.31	3.5 ± 2.8	2.6 ± 2.6	−0.34
Hip adduction (°)	9.4 ± 8.7	6.1 ± 4.2	−0.49	6.4 ± 5.3	4.9 ± 2.9	−0.51
Knee adduction (°)	9.5 ± 7.7	12.3 ± 7.9	0.36	19.0 ± 13.2^a^	19.7 ± 12.3^b^	0.05
Hip abduction (°)	7.4 ± 6.3	5.7 ± 5.8	−0.28	7.8 ± 7.5	11.1 ± 8.1^b^	0.42
Knee abduction (°)	4.2 ± 2.6	2.2 ± 1.6	−0.89*	7.8 ± 6.3^b^	2.3 ± 2.2	−1.18*
Trunk lateral tilt (°)	5.3 ± 4.8	8.2 ± 4.4	0.63*	4.3 ± 2.4	3.6 ± 3.0^a^	0.23

*indicates significant difference between limbs at *p* < 0.05

^a^indicates large within-limb effect size between environments at Cohen’s *d* > 0.8

^b^indicates moderate within-limb effect size between environments at Cohen’s *d* > 0.8

Water immersion also affected the degree of asymmetry in both groups during the exercises, as was indicated by the SI-scores (Table 3). The SI analysis revealed that the groups were affected differently by the changed environment, although no obvious trends indicated whether water immersion increased or decreased the degree of symmetry in either group. For example, during the SLS, the AKP group had increased SI-scores for hip flexion and decreased scores for anterioposterior trunk motion in water. Meanwhile, the control group showed increased SI-scores for knee and hip abduction during DLS, and reduced scores for hip abduction during the SLS when the exercises were performed in water.

**Table 3:** Asymmetry index score (±SD) between the groups in both environments

		Land			Pool		
		AKP	Control	*d*	AKP	Control	*d*
Double-leg Squat							
	Shank AP (°)	5.9 ± 1.1	5.4 ± 1.2	0.40	5.9 ± 1.6	5.8 ± 1.4	0.06
	Thigh AP (°)	5.3 ± 0.7	5.0 ± 1.4	0.33	5.3 ± 0.5	5.1 ± 1.7	0.12
	Knee flexion (°)	0.3 ± 0.1	0.4 ± 0.4	−0.62*	1.3 ± 1.3^a^	0.5 ± 0.5	0.76^a^
	Hip flexion (°)	1.4 ± 0.8	1.3 ± 0.7	0.11	1.6 ± 1.1	1.7 ± 1.7	−0.09
	Shank ML (°)	1.9 ± 1.3	2.3 ± 1.7	−0.26	2.7 ± 2.1	2.2 ± 2.7	0.20
	Thigh ML (°)	2.9 ± 2.1	3.0 ± 2.2	−0.06	3.6 ± 2.0	3.1 ± 2.2	0.24
	Knee abduction (°)	2.2 ± 2.0	4.8 ± 1.5	−1.48*	3.3 ± 2.5	6.0 ± 1.7^b^	−1.26*
	Hip abduction (°)	2.8 ± 2.0	4.6 ± 2.1	−0.91*	3.0 ± 1.9	7.4 ± 2.6^a^	−1.87*
Single-leg Squat							
	Shank AP (°)	5.3 ± 1.4	5.1 ± 1.3	0.15	6.3 ± 1.7	6.3 ± 1.3^a^	−0.13
	Thigh AP (°)	4.9 ± 0.8	5.0 ± 0.8	−0.13	4.8 ± 0.9	5.0 ± 0.9	−0.21
	Thorax AP (°)	4.3 ± 2.1	1.8 ± 1.3	1.48*	2.0 ± 1.4^a^	2.0 ± 1.7	0.02
	Knee flexion (°)	0.7 ± 0.5	0.7 ± 0.4	0.07	0.7 ± 0.4	2.0 ± 1.7^a^	−1.05*
	Hip flexion (°)	2.2 ± 1.0	1.5 ± 0.6	0.85*	1.3 ± 0.7^a^	1.4 ± 0.8	−0.14
	Trunk flexion (°)	3.4 ± 1.9	2.3 ± 1.2	0.69*	2.4 ± 1.5^b^	2.3 ± 1.5	−0.04
	Shank ML (°)	7.0 ± 5.7	2.7 ± 1.6	1.04*	2.4 ± 1.1^a^	2.6 ± 2.0	−0.13
	Thigh ML (°)	7.1 ± 4.7	3.5 ± 2.4	0.96*	2.8 ± 1.4^a^	3.9 ± 2.0	−0.66*
	Thorax ML (°)	15.3 ± 14.9	6.1 ± 4.6	0.84*	5.3 ± 4.0^a^	5.8 ± 3.8	−0.13
	Knee abduction (°)	8.4 ± 7.3	5.0 ± 1.8	0.64*	7.1 ± 3.1	6.0 ± 2.9	0.37
	Hip abduction (°)	7.3 ± 5.6	8.2 ± 6.7	−0.14	7.3 ± 4.6	4.7 ± 2.8^b^	0.70*
	Trunk lateral tilt (°)	8.1 ± 8.6	5.1 ± 3.0	0.45	11.5 ± 9.4	6.6 ± 4.1	0.67

*indicates significant difference between environments at *p* < 0.05

^a^indicates large within groups effect size between environments at Cohen’s *d* > 0.8

^b^indicates moderate within groups effect size between environments at Cohen’s *d* > 0.5

The SI scores also differed between the groups. Although the analysis often indicated higher scores for the AKP in both environments, the control group showed more asymmetry in knee and hip abduction during both land- and water-based DLS. They also showed higher SI-scores for knee flexion during water-based SLS.

## Discussion

Primary findings from this study were that participants with AKP employed different kinematics in the affected and unaffected limbs during DLS and SLS performed on land and in water. Immersion appear to increase kinematic differences between the limbs, perhaps because of the more dynamic environment [29]. Further, although the aquatic environment seemingly affected the SI-scores both in individuals with AKP and in uninjured controls, the analysis showed no obvious trends towards more or less asymmetry in either environment.

The results from this study suggested that water immersion allows individuals with AKP to achieve greater squat depth during both DLS and SLS, compared to when performing the exercises on land. The increased depth during the SLS was reflected in increased peak hip and knee flexion angles. During the DLS, increased depth occurred most likely due to several non-significant increases in joint angles in the lower body. The reduced loading in water no doubt allowed greater movement depth without resulting in discomfort or pain at the knee. Water immersion can therefore improve knee joint range of motion during squat tasks in this population. Re-establishing knee joint range of motion is a primary goal in early rehabilitation for AKP [1], and practitioners are encouraged to recognize the benefits of increased squat depth during rehabilitation for this population.

Interestingly, the AKP group showed similar peak angles during land-based DLS in both limbs in the sagittal and frontal motions. These observations support previous research that reported comparable flexion angles during DLS in individuals with previous ACL injury [11, 30]. However, the authors stressed that kinetic differences existed between the limbs, and cautioned that compensatory movements may not be reflected in the kinematics. The authors also highlighted that it often is difficult for practitioners to identify joint substitutions without access to kinetic measurements. It is possible the AKP group in this study employed compensatory movement strategies that would have been evident on land during kinetic assessments, despite appearing symmetrical during the kinematic analysis.

Kinematic differences appeared between the limbs during DLS performed in water that were not evident on land. Interestingly, while the unaffected limb appeared to maintain its kinematics in both environments, increased hip abduction in the affected limb indicated a wider stance in water, while the body remained over the unaffected limb. Perhaps this strategy indicated a shift in loading towards the unaffected limb, but kinetic analyses are needed for confirmation. Previous research has suggested that water immersion changes balance demands [18, 19], and the wider stance was perhaps a balance strategy. However, the effects of changed balance demands on exercise performance and outcomes has not been well-documented in the literature. Research on effects of water immersion on kinematic symmetries is scarce, but similar to the results from this study, increased asymmetries has been reported by one previous study [22]. The authors suggested that the increased asymmetry probably reflected pre-existing functional differences due to greater instability in water. It is possible the asymmetries that appeared during water-based DLS were reflections of compensatory movement strategies revealed by the aquatic environment.

The gravitational offloading [14], decreased pain [31], and altered proprioception [19] in water likely changed the demands of the exercises, perhaps to the extent where established movement strategies were disrupted and asymmetries were revealed. Currently, not enough research has been conducted on the topic to determine whether the different kinematics between the affected and unaffected limbs in water were associated with compensatory strategies. Future research should assess kinetic profiles and quantify environmental effects on compensatory movements. The possibility that water immersion may reveal existing kinematic differences is exciting as it provides practitioners with a useful movement assessment tool that is not currently available.

Kinematic differences between the limbs also existed during the SLS in both environments and were evident both the sagittal and frontal planes of motion. Land-based SLS on the affected limb showed decreased hip flexion, increased varus alignment and lateral trunk lean. The reduced hip flexion probably indicated a strategy with less hinging from the hip, which shifts the centre of mass posteriorly, and reduces the demand of the gluteal muscles [10]. This increases the demand of the quadriceps, and consequently the compressive loads of the patellofemoral joint [32], which potentially contributes to the continued aggravation of AKP. Research has also suggested this might contribute the weak gluteal muscles that are often reported in this population [7, 10]. The compensatory movements employed by the AKP group on land may therefore aggravate their condition further. Importantly, this adaptation was not reduced in water, despite the considerable offloading.

The increased lateral trunk lean on land during SLS on the affected limb supported previous research that reported increased frontal plane movements in individuals with AKP [2, 9]. The reduced trunk lean in water suggested that immersion may provide some support to the trunk.

Our results showed marginally reduced valgus alignment on the affected limb, although previous research has reported increased valgus in this population [2, 9]. The previous authors suggested that increased valgus was associated with hip-muscle weaknesses. The reason for this discrepancy with previous research remains unknown, but the knee abduction angles in the unaffected limb were similar to previous reports for healthy controls [9]. Interestingly, water immersion increased the knee abduction angles of the affected limb, while it increased the varus alignment in the affected limb. This was likely a positive observation, as increased valgus alignment is associated with decreased functionality and injury [3, 33]. Further research is needed to determine the functional effects of these frontal plane adaptations during the DLS.

The increased hip abduction angles during water-based SLS cannot be attributed to a wider stance, as it is unilateral exercise. Previous research have suggested that increased balance demands in water requires an increased reliance on frontal plane motions [20]. Increased lower body motions in the frontal plane is perhaps a normal response to the unstable nature of the aquatic environment. This study did not quantify balance so the implications of water immersion on postural control remain unknown, however, previous research reported improved land-based balance following water-based training [19, 34]. Although these studies did not measure balance during immersion. Research has reported increased postural sway in water during quiet standing [35], but did not assess dynamic movements. Future research should analyse ground reaction forces and perturbations in centre of pressure during water-based exercises to further the understanding water immersion on balance strategies.

The SI analysis showed that water immersion often affected bilateral asymmetries in both individuals with AKP and healthy controls. Regardless, practitioners should acknowledge that some asymmetry is normal even within a healthy population [22–24], although research is yet to determine the threshold for when asymmetrical movements should be considered undesirable. The SI-scores in this research ranged from 0.3 to 15.3, and researchers using the same SI method reported scores between 8 and 16, but did not refer to whether this should be considered normal [24]. Therefore, the practical implications of these values remain unclear.

Some SI-scores indicated more asymmetry on land, while others suggested more asymmetry in water. The observations of increased SI-scores in water agree with previous research [22], however, the implications of this are still unknown. Practitioners should consider that the emphasized asymmetries in water may be detrimental for rehabilitation. Asymmetrical motor patterns can reduce the efficacy of rehabilitation exercises [11], which highlights the need for close monitoring during rehabilitation. Further, prolonged asymmetrical motions at the knee joints has been suggested to increase the risk of osteoarthritis [11]. However, it is possible that gravitational offloading in water reduces long-term implications of asymmetrical loading. Additionally, the participants in this study had no prior experience with water-based exercise, so it is possible that habituation could change these results and reduce the degree of asymmetry during the water-based exercises. Regardless, the offloading constitutes a primary rationale for employing aquatic therapy for rehabilitation [16] as it allows for earlier return to partially loaded activities. Continuous movement assessments throughout a rehabilitation program can highlight asymmetries and potentially indicate the efficacy of the program.

Researchers have highlighted lacking understandings on implications of water immersion on movement symmetry [22], which deserves attention in future research. This study assessed kinematic effects of water immersion, and future research is still needed to assess the effects of water immersion on kinetic and neuromuscular profiles of individuals with AKP. This would provide practitioners with a clearer understanding of the roles of water-based rehabilitation for this population. Further, the transferability of movements between the environments has not been established and it is possible that any beneficial movement adaptations observed in water is confined to pool-settings. This necessitates that future research determines the degree of transferability between water and land to optimize current guidelines for practitioners.

## Conclusions

Water immersion allowed individuals with unilateral AKP increased depth during DLS and SLS, along with some increased flexion angles. The increased movement range caters to early rehabilitation goals for individuals with AKP. The exercise environment also affected the movement patterns differently between limbs. The degree of asymmetry was affected in both groups during the exercises, although the long-term implications of this remain unknown. Increased asymmetries during water-based exercises suggests that clinicians should pay close attention to their client’s technique and perhaps use verbal and visual feedback to minimise any movement compensations. This study suggests that practitioners should consider aquatic therapy as one component of a comprehensive treatment plan for participants with long-standing AKP, and use it in conjunction with established protocols.

## Abbreviations

AKP: Anterior knee pain
DLS: Double-leg squat
SI: Symmetry index
SLS: Single-leg squat
